# ‘If it’s a medical issue I would have covered it by now’: learning about fibromyalgia through the hidden curriculum: a qualitative study

**DOI:** 10.1186/s12909-017-0972-6

**Published:** 2017-09-12

**Authors:** V. Silverwood, C.A. Chew-Graham, I. Raybould, B. Thomas, S. Peters

**Affiliations:** 1 Research Institute for Primary Care and Health Sciences, Keele University, Newcastle, Staffordshire ST5 5BG UK; 2 West Midlands CLAHRC, Westminster, Staffordshire UK; 3 Manchester Centre for Health Psychology, School of Health Sciences, Manchester University, Manchester, M13 9PL UK

**Keywords:** Fibromylagia syndrome, Undergraduate medical students, Attitudes and perspectives, Medical curriculum

## Abstract

**Background:**

Fibromyalgia syndrome (FMS) is a long-term condition that affects between 1 and 5% of the general population and lies within the spectrum of medically unexplained symptoms (MUS). FMS can be difficult to diagnose and is usually done so as a diagnosis of exclusion. There is continuing debate regarding its legitimacy excluding other causes of symptoms. It is known that the diagnosis and management of MUS, including FMS, receives little attention in medical curricula and attitudes towards patients with FMS amongst medical professionals and trainees can be negative. The purpose of this study was to investigate how attitudes and perspectives of undergraduate medical students towards FMS are acquired during their training.

**Methods:**

Qualitative interviews with 21 medical students were conducted to explore their views on FMS, encounters with patients with FMS, and where learning about FMS occurs. Participants were recruited from two English medical schools and the study was approved by two University Ethics committees. Interviews were digitally recorded with consent and data analysed thematically, using principles of constant comparison.

**Results:**

The data were organised within three themes: i) FMS is a complex, poorly understood condition; ii) multiple sources for learning about FMS; and iii) consequences of negative attitudes for patients with FMS.

**Conclusion:**

Undergraduate medical students have limited understanding of, and are sceptical over the existence of FMS. These attitudes are influenced by the ‘hidden curriculum’ and witnessing attitudes and actions of their clinical teachers. Students interpret a lack of formal curriculum teaching around FMS to mean that it is not serious and hence a low priority. Encountering a patient, friend or family member with FMS can increase knowledge and lead to altered perceptions of the condition.

Teaching and learning about FMS needs to be consistent to improve knowledge and attitudes of clinicians. Undergraduate students should be exposed to patients with FMS so that they better understand patients with FMS.

## Background

Fibromyalgia syndrome (FMS) is a chronic condition first described in the 1970s [1]. Diagnostic criteria describes fibromyalgia in terms of four areas of clinical features: chronic widespread pain (CWP), sleep disturbance, fatigue and bilateral tender points around the body [2]. The American College of Rheumatology approved clinical diagnostic criteria and a measure of symptom severity, which are based on patients’ symptoms [3]. FMS affects between 1 and 5% of the general population [4] and lies within the spectrum of ‘Medically Unexplained Symptoms’ (MUS) conditions, where there is no underlying biomedical explanation for the symptoms.

There is continuing debate regarding its legitimacy as a diagnosis [5]. This may be because the diagnostic criteria may be viewed as arbitrary [6] or because there is lack of clear pathophysiological explanation [7]. FMS can also be associated with a variety of symptoms, creating potential for diagnostic uncertainty. In addition, there is overlap of symptoms with other conditions such as Irritable Bowel Syndrome (IBS) and Chronic Fatigue Syndrome (CFS). Patients who have symptoms suggestive of FMS report difficulties in their interactions with healthcare professionals [8]. Clinicians admit to negative perceptions of patients with FMS [9] and this creates communication problems with both patient care and doctor-patient relationships. One study found that up to a quarter of General Practitioners (GPs) felt that those with FMS were ‘malingerers’ [10], meaning that their impression of FMS patients is already formed prior to consultations with patients suffering from FMS.

FMS is often a diagnosis of exclusion; patients are likely to have undergone investigation for other conditions with no pathology identified [11]. However, it has been suggested that the positive examination finding of tender points should help clinicians view FMS as a legitimate, acceptable diagnosis [8]. There are a number of reasons why clinicians are reluctant to make a diagnosis of FMS and it has been suggested that clinicians do not possess the necessary competencies to make a formal MUS diagnoses [10, 12, 13]. Training GPs to support and manage people with MUS can help to improve the patient’s experience during a consultation and help clinicians explain MUS to patients [14, 15]. Unfortunately, there is limited availability of such training [16] and GPs with more negative attitudes towards MUS may decline additional training about FMS [17].

Despite its prevalence, the diagnosis and management of MUS, including FMS, receives surprisingly scant attention within medical curricula [18]. Despite the lack of training, students still develop beliefs, including negative views, about patients, and the diagnosis and management of MUS, though it is not clear how these views are acquired [19].

Therefore, the aim of this study was to investigate the acquisition of UK undergraduate medical students’ learning about FMS.

## Methods

This study was set in two English medical schools both of which use Problem Based Learning (PBL) as a learning approach. PBL is a group work approach that involves learning via ‘cases’; using clinical reasoning skills and identifying appropriate learning needs and objectives via an interactive process [20]. University Ethics approval was granted by both institutions.

### Study design

Semi-structured interviews were used to explore students’ perceptions of FMS. This provided unrestricted focus on the research question with freedom to explore emerging issues with participants. None of the researchers conducting the interviews had any role in educating the students being interviewed.

### Quality assessment of methods

The qualitative research quality tool ‘Consolidated criteria for reporting qualitative research checklist’ (COREQ) [21] was referred to as a method to assess the quality of the methods in our study. This tool prompts researchers to assess areas such as the theoretical framework, participant selection, data collection methods and data analysis amongst others.

### Sampling and recruitment

The only inclusion criterion was that students were currently enrolled on the undergraduate medicine course at either university (including students working towards intercalated degrees, when a student takes a year between medical studies to complete an additional degree). A recruitment email was sent to all undergraduate medical students inviting them to take part, and supplemented with posters advertising the study. From those who responded, purposive sampling was used to ensure representation of each year group of the undergraduate degree and a variance between gender and age. Students were given a shopping voucher of £10 to thank them for their contribution.

### Data collection

After discussion and review of the literature, a topic guide was developed by the research team, which explored students’ views on FMS, diagnosis and management of patients and experience of learning about FMS. Interviews were conducted until data saturation was achieved (the point at which no new ideas relevant to the research question emerged). The interviews were conducted by one of three authors (VS, BT, IR), face-to-face with participants in university buildings. Participants were provided with a Participant Information Sheet, and asked to give written consent before participating. The interviews were digitally audio-recorded and transcribed verbatim. Identifying information (e.g. names and places) was removed.

### Data analysis

Analysis was guided by the principles of the constant comparative method [22, 23]. Codes were identified in the early interviews and the topic guide was amended to allow exploration of emerging ideas in subsequent interviews and seek contradictory data. Transcripts were read by at least two authors and a subset read by all other authors. Codes were revised and refined until the thematic structure identified. Any coding inconsistencies were resolved through discussion.

## Results

Twenty-one undergraduate medical students from two English medical schools were interviewed. Table 1 provides participant demographic information. Mean interview length was 27.45 min (range = 16.47–42.14).

**Table 1:** Participant demographic information

Participant number	Year of degree	Age range (18–23, ≥24)	Gender	Ethnicity	Declared personal knowledge of FMS outside of medical school	Interview length (mins:secs)
1	4	≥24	F	Indian	Yes	17:22
2	2	≥24	M	Australian Caucasian	No	23:27
3	Intercalating^a^	18–23	F	British Caucasian	No	38:28
4	5	≥24	M	British Caucasian	No	23:06
5	Intercalating^a^	≥24	M	British Caucasian	No	27:16
6	1	≥24	F	British Caucasian	No	36:28
7	3	≥24	M	British Caucasian	Yes	42:14
8	1	18–23	M	Bengali	Yes	16:47
9	3	≥24	F	Afro-Caribbean	Yes	27:05
10	3	18–23	M	British Caucasian	No	28:47
11	1	18–23	F	British Pakistani	Yes	33:20
12	5	≥24	F	British Caucasian	No	29:34
13	5	≥24	M	British Caucasian	No	25:23
14	5	≥24	F	British Caucasian	No	25:00
15	4	18–23	F	British Asian	No	25:41
16	Intercalating^a^	18–23	M	British Caucasian	No	27:28
17	2	18–23	F	British Caucasian	Yes	29:15
18	2	18–23	F	Indian	No	29:40
19	2	18–23	F	British Caucasian	No	13:24
20	3	18–23	F	British Caucasian	Yes	32:40
21	3	≥24	F	British Caucasian	No	27:45

^a^Intercalating is when a student takes a year between studies to complete an additional degree, usually taken between the fourth and fifth year of studying for an undergraduate medical degree

The data were organised within three themes: i) FMS is a poorly understood, complex condition; ii) Multiple sources of learning; and iii) consequences of negative attitudes for patients with FMS. Illustrative data is used to support the themes, labeled with respondent identifier.

### FMS is a poorly understood, complex condition

Students viewed FMS as a complex condition, with an uncertain aetiology that was likely to involve multiple factors. They suggested that FMS is a poorly understood condition.

*‘I would say it’s probably very complex disease that’s um, lots of different precipitating factors that interplay’* (ID3).

All but one students disclosed uncertainty or were tentative about describing FMS in any detail. The exception was still largely descriptive:

*‘So I think it’s a syndrome of chronic, persistent pain, that isn’t attributed to another known organic pathology, associated with particular tender spots on the body, based on the concept of central nervous system hyper-sensitisation.’* (ID13).

Some students recognised that current scientific knowledge is lacking on the condition and believed that, as medical science progresses, we may develop greater understanding about FMS:

*‘It’s one of those conditions which sort of exposes the limitations of modern medicine’* (ID2).

*‘I guess I’d see it as a blind spot that’s slowly being chipped away at*. *.*. *but then I think there are lots of things that we don’t actually understand about medicine, there are definitely some areas of mystery still around.’* (ID13).

Respondents recognised that it could be a lengthy and difficult process for patients to be diagnosed with FMS. They suggested that as there was no specific diagnostic investigation that would confirm the diagnosis, FMS was a ‘diagnosis of exclusion’, and that efforts should be prioritised to rule out organic causes first:

*‘So all I do know about it is that doctors- it's kind of a diagnosis of exclusion. It's something that, um, there's not a test for. It's not something that's easy to diagnose because there's, like I said, there's not a test for it and it can be quite different for every patient’* (ID 14).

Respondents varied in their views about how FMS should be classified. Areas suggested were musculoskeletal, neurological and psychiatric/psychological conditions. As a consequence, students were unsure where this condition fitted in their learning and what the best approaches for management might be:

*‘But I don’t think there is any consensus as to whether it’s psychological or physical, I don’t think. I mean, maybe it’s a mix of mental and biomedical.’* (ID8).

### Multiple sources of learning about FMS

Students described a variety of sources for their acquisition of learning about FMS: the medical curriculum including both explicit and hidden elements, clinical role models and the experiences and perspectives of friends and family.

#### The medical curriculum

Students suggested that FMS was not explicitly covered within the undergraduate curriculum; and opportunities for learning that students’ recalled were limited:

*‘We learnt about it in our fourth year, none of it was in great detail, it was just like, one of those little things, that kind of we discussed a bit, but not a lot’* (ID 12).

Because of the lack of specific teaching about FMS, the ‘hidden curriculum’ was influential; with students interpreting lack of formal teaching to mean FMS is not important, or even a legitimate medical problem:

*‘Yeah… I think if it was medical issues- If I, I might have got completely the wrong end of the stick, but I think if it was a medical issue I would have covered it by now’ (ID 14).*

Its scant inclusion led students to infer that it was less serious or important than other conditions, which should therefore take priority compared to patients with FMS:

*‘perhaps with no teaching on FMS, then you think that it’s not as prominent or not as important to learn about.’* (ID 3).

*‘I don’t think you see enough of it for it to take a big part of the curriculum up in comparison to other things like chronic heart disease or renal failure*. *.*. *stuff like that is very important.’* (ID5).

However, other students suggested that *because* FMS was complex it should be covered explicitly:

*‘For a condition that we don’t understand well and that there aren’t any nice laid out guidelines for how to treat that would seem to be the sort of condition that we ought to talk a bit more about.’* (ID4).

They considered that lack of awareness and understanding about FMS could be problematic; if medical students are not aware of the condition then as qualified doctors they are not likely to make the diagnosis confidently, or understand a patient’s concerns:

*‘I think there is a general lack of training about it, I think doctors do what they can but they don’t seem to know very much about it.’* (ID11).

#### Learning from the patient

Students did not want to be taught about FMS exclusively through lectures. Adjunctive learning from patients was described as offering the possibility of students understanding the problems faced by individuals with FMS, not just in their experience of symptoms, but also discussion of interactions with clinicians.

*‘I don't see the point of a case they give to us and then they don't know what the cause is? We can't really put things in perspective. So I think it's best when we see a patient talk about it, we learn about the symptoms, and we go out and we're interested, like, 'Oh, what can be causing this?’* (ID 18).

#### Learning from friends and family

Several respondents disclosed that they knew somebody in their personal life, (such as a friend or family member), who had been diagnosed with FMS and discussed how this had offered a different perspective about the condition. Some students suggested that their sole source of learning about FMS was through discussion with this individual, rather than any formal teaching:

*‘If I hadn’t had any personal experience of it* [family member diagnosed with FMS] *I don’t think I would really know anything about it*. *.*. *And I think that like having a sort of personal experience of it, I’m already like miles ahead of everyone else. I’d say the majority of my colleagues wouldn’t really know the first thing about it to be honest.’* (ID20).

Students reported that having this personal experience allowed them to appreciate the problems faced by those with FMS. They also could apply this increased level of knowledge to other conditions, meaning they felt they were less judgmental with other patients:

*‘I think having personal experience* [family member diagnosed with FMS] *of it means that I view it very differently from other medical students or even doctors, I think I’m more open minded and that you need to be careful about judging patients and dismissing their symptoms as being “all in their head”, I think that’s a really disparaging term that doesn’t help the patients or the healthcare professionals.’* (ID11).

Some students expressed concerns that as FMS wasn’t covered in the curriculum explicitly it was seen to be unimportant by their colleagues, and only because of their personal experience, did they recognise it as an important condition:

*‘I think maybe for me, because I’ve heard of it through my [relative] I think it’s something important and they could definitely touch on it a bit earlier on’* (ID7).

#### Clinical role models

Although FMS was not necessarily covered explicitly in the curriculum, students reported learning about the condition through contact and observation of clinical teachers, who could be influential on students’ understanding of, and attitudes towards people with FMS. They felt reluctant to question unfavourable views expressed by those in senior positions who taught them:

*‘Now, I think, I think because as a student you’re very much in a bubble that anybody who is qualified, at whatever level, is superior, and they must know the answers and what they say is right. They’re very influential.’* (ID3).

*‘Well, I suppose if somebody teaches you about a condition, you can’t help but be influenced by them, they’re qualified and you kind of sometimes take on their opinions’* (ID10).

Students reported many instances of having encountered negative attitudes towards FMS patients from clinical colleagues:

*‘*“*It’s all in their head”*. *.*. *I suppose it’s just a very disparaging term for, it’s a ‘psychological condition’*. *.*. *a lot of clinicians might feel that it’s ‘all in their head’ rather than a physical condition.’* (ID9).

*‘I don’t think as a doctor of the future I would, I don’t know, judge patients just by saying that they’re putting it on, or at least I hope I won’t.’* (ID7).

Students reported that their peers, on occasions, had expressed negative attitudes towards patients with FMS, with the suggestion that as students became more senior their sense of understanding and empathy towards people with MUS reduced:

*‘I think as we’ve gotten further and further along in the course, people seem to be generally less empathetic, and I mean there are some people that aren’t like that, but there are some people who, you just think, “ooh, why are they like that?” it’s like they’ve become jaded.’* (ID12).

Rare reports of positive attitudes towards people with FMS from their clinical teachers were attributed to the individual clinician rather than the training system:

*‘I was only with him [the GP] for that session, um, but he was knowledgeable*. *.*. *he seemed to know what he was talking about and I suspect that he’s probably come across it quite a lot and kind of learnt on the job maybe, I don’t know’* (ID 21).

### Consequences of negative attitudes for patients with FMS

Students could clearly identify the negative consequences of the inadequacies of medicine and medical training on patients’ experiences. They had viewed uncomfortable encounters between patients and clinicians and heard first-hand about the effort that patients had to take to be taken seriously. Participants felt that patients had to convince healthcare professionals that their symptoms were genuine in order to receive acknowledgement of them:

*‘But it was a massive struggle for her to get recognition, from the time she first went to a doctor to the time that occupational health actually said, “yes, it is fibromyalgia,” there was a big fight to get recognition of it.’* (ID7).

*‘It's quite sad, they tend to, to almost, I get the feeling they don't think people take them seriously, they're almost pleading to be taken seriously in a lot of cases’* (ID 16).

They also reflected on how the uncertainty around the condition could cause doctors to feel frustrated about their lack of knowledge around diagnosis and management of FMS which impacted negatively on patient’s experiences:

*‘I think like, a lot of the time, not all doctors, but if they can’t do anything for it, it kind of tests them, and they don’t feel happy because they’re like to be able to fix everything and then I think that might get taken out on the patient, you know, their own feelings of inadequacy*. *.*. *I’ve sat there and felt like that has been the case before.’* (ID12).

They suggested that patients’ problems might be dismissed by their doctors, if no organic cause for pain was found after repeated investigations:

*‘And I think… they, because they, it's not really well-understood they don't really explore the patients' concerns and sometimes they don't also recognise that the Chronic Widespread Pain is really quite serious. They just think it's, because there's so many, y'know… So many patients that present with pain, that it's just another patient who wants medication…Y'know they just medicate them as opposed to treating them holistically.’* (ID 15).

Students discussed their concerns that if they, as undergraduates, did not develop a coherent understanding of FMS then they would face challenges as doctors, similar to those they had observed. Moreover, that training for existing doctors was needed to improve current care and supervision. Ultimately, students believed that the focus of the problem should be on improving understanding of FMS:

*‘And prevent people from turning into doctors that don't understand it and therefore just have an opinion. Most wrong. … most questionable opinions are based on a lack of understanding’* (ID 14).

## Discussion

### Summary of results

This is the first qualitative study to examine the perspectives of UK undergraduate medical students and how they acquire knowledge about FMS. Students reported a lack of understanding about the condition, diagnostic process and optimal management. They suggested that FMS is not covered in the undergraduate medical curriculum because there is insufficient understanding of its’ pathophysiological aetiology. Students inferred that the absence of teaching around FMS means it is a rare condition, not serious, and maybe not a legitimate medical problem to diagnose and manage. In fact, FMS has similar prevalence to type two diabetes mellitus in the general population [24], yet, unlike diabetes, is largely absent from the formal curriculum. This means students draw their inferences about the condition from lack of teaching and the ‘hidden curriculum.’ This has implications for patient care: if students have limited understanding of FMS, then as doctors, this is unlikely to change. This highlights both a need for further clinical research into FMS and also an educational need for increased teaching and learning both within undergraduate and postgraduate educational settings.

Some students discussed personal contact with an individual diagnosed with FMS. These students reported a broader understanding of the condition and perceived themselves to have more positive attitudes around FMS and those diagnosed with it.

Students reported being influenced by the explicit curriculum but also ‘hidden’ elements, particularly witnessing actions and attitudes of clinical role models, which proved highly influential. First described by Hafferty in 1994 [25], the hidden curriculum is common within medical schools. It includes the communication of culture from teacher to student within both formal and informal teaching environments. The hidden curriculum can be very influential; ‘playing an important role’ in developing a ‘professional identity’ [26]. Mahood describes the hidden curriculum as a ‘socialisation process,’ whereby students are affected by implicit teaching within everyday examples from clinicians [27]. It has been suggested that the hidden curriculum could be detrimental in some ways as students may become more ‘cynical and insensitive’ towards patients [28]. Hopkins suggests it could even be ‘empathy disabling,’ as students observe and subsequently adopt potentially negative attitudes and behaviours learnt from clinicians [29].

Students reported seeing clinicians who were dismissive of patients and speculated that this could be due to their lack of knowledge or negative personal attitudes about FMS. Although they were influenced by this, students also reported that they were reluctant to question experienced clinicians’ views. The educational concept of andragogy, first described by Knowles twenty-five years ago, assumes that adult learners become self-directed and are able to both determine their own learning needs and find means to achieve their educational goals [30]. Knowles was an advocate for encouraging students to question their teachers; something which our data demonstrates does not occur [30]. Miller & Schmidt suggested that many clinicians are not fully aware of this ‘unconscious influence’ upon students [31]. They advised that clinicians should take care to ensure that they practice altruistically, especially when they have medical students observing them who may be influenced by their behaviour.

### Comparisons with wider literature

In this study, students suggested that, due to the current lack of scientific evidence and understanding of FMS they had significant knowledge gaps and felt that learning directly from patients helped them to in order to understand the true patient experience. This knowledge gap is demonstrated to persist beyond undergraduate training. Yon et al. found that junior doctors feel incompetent in working with patients with medically unexplained symptoms and frustrated and anxious by this [32]. Without a more helpful model, they categorise patients as malingers or mentally ill. Maatz et al. discussed the concept of ‘difficult’ to treat MUS patients and the negative reactions that can occur when secondary care physicians face these patients and feel frustrated and unable to help them [33].

Despite students expressing frustration about the general lack of understanding of FMS, none questioned the validity of FMS as a diagnosis. This differs from the general medical community where scepticism of the diagnosis is still present [5]. Similar findings were presented by Amber et al. [34], who found that medical students in the US were more likely to support a physiological mechanism for FMS than qualified physicians. They suggested this difference in perceptions could be due to FMS becoming more ‘readily accepted’ amongst the medical community as a legitimate diagnosis or that scepticism is more common amongst qualified clinicians.

A study looking at Canadian complementary and alternative (CAM) healthcare professionals (including physiotherapy, chiropractic and occupational therapy students) views of FMS also demonstrated that they felt FMS was a legitimate diagnosis and that effective therapy for FMS exists [35]. This suggests that healthcare professional students are generally accepting of FMS as a clinical entity. Internationally, CAM is used by the majority of FMS patients [36]. This suggests that CAM students are frequently exposed to FMS; therefore, they demonstrate increased acceptance and greater understanding of how to manage the condition.

Medical students in this study described a variety of sources of knowledge about FMS; learning from friends, family and personal experiences. Previous studies have shown that students with personal experiences of illness are more likely to have improved attitudes and empathy towards patients [37]. This inclination to seek alternative information sources also occurs with clinicians as demonstrated by Chew-Graham et al. [12]. They found that even experienced doctors recognise that their medical training does not equip them with the skills and knowledge to diagnose and manage CFS hence they seek alternative sources.

In this qualitative study, we did not investigate a link between seniority of the medical student and their understanding of FMS, and we did not assess accuracy of their knowledge or understanding; rather, we aimed to explore medical students’ perceptions and experiences of patients with FMS. Amber et al. reported that as medical students became more senior they felt more confident about their knowledge of FMS but this didn’t necessarily correspond to true understanding [38].

Students in this UK study were unsure about the aetiology of FMS, which contrasts to a US study where direct-to consumer advertising appeared to affect medical students’ perception of their understanding of FMS. This study suggested that pre-clinical US medical students, who had not received formal teaching about pathology of FMS, could be influenced by consumer advertising [39]. This is not an issue affecting UK medical students because in the UK pharmaceutical consumer advertising does not occur. The extent to which advertising is a source of information and how this should be managed is certainly a possible avenue for further research to explore. However, this contrast demonstrates that internationally there could be significant differences in the factors influencing medical students understanding of FMS.

Studies exploring medical students’ and junior doctors understanding of medically unexplained symptoms (MUS) [32] reported that students had developed ideas about MUS despite not having received any formal training, and interactions with clinical teachers were particularly influential in shaping students’ attitudes. As previously discussed, the ‘hidden curriculum’ played an important part in development of thoughts and attitudes towards FMS and seems to dominate learning about the condition [40]. It has been recommended that any training needs to be evidence-based and clinically relevant [19]. A previous study looking at chronic pain and depression also suggested that early and continued education contributes to a reduction in negative attitudes towards patients, something which is also supported by the views discussed in this study [41].

### Strengths and limitations

This is the first study to explore qualitatively the attitudes of medical students about FMS, a common and disabling medical condition, and the impact of the hidden curriculum on medical training about FMS. Data saturation was achieved. Data were analysed by a research team with members of different professional backgrounds, a recognized method to increase trustworthiness of the analysis [42]. This study was conducted in two UK medical schools, both utilising PBL curricula; thus the results may not be generalisable across all English medical schools.

Students chose to take part in the study and those that did volunteer are more likely to have personal interest in the study and personal experience of a patient or family member with the condition. Students were given a voucher as an inducement to be interviewed and it was asserted the interview was not a test of their knowledge or experience, which may have mitigated against this to some extent. However seven respondents reported personal knowledge of a person with FMS supporting previous findings that doctors take part in research which is personally interesting to them [17]. Therefore, our sample is likely to *under-represent* negative attitudes towards FMS.

### Recommendations for curricula and future research

Explicit and evidence-based teaching and learning about MUS, such as FMS is needed in order to increase knowledge and confidence of clinicians and thereby improve patient care. Students should ideally be exposed to patients with FMS during their undergraduate studies so that they understand this symptom complex and the challenges of making a diagnosis in conditions where there is no diagnostic test. Students have been shown to be receptive to learning from patients, reporting that they learn realistic attitudes from patients who suffer from chronic disease [43]. Medical schools could facilitate interaction with patients who suffer from FMS to discuss their condition with students; encouraging learning about the condition and consultations with health professionals. There is evidence that patient educators can be as effective as senior clinicians in explaining conditions, including musculoskeletal disorders [4], and they are increasingly involved in delivering training [3].

However, given that a core barrier found was attitudes of clinical tutors, we suggest that the ethos of the learning environment needs to adapt. Students should be actively encouraged to engage clinicians and tutors in professional discussion about clinical presentations where uncertainty exists, as often occurs in FMS patients. The clinical learning environment should also be as supportive as possible to encourage students to feel comfortable enough to question negative attitudes of health professionals.

Further research is needed investigate the impact of training packages about FMS, but also MUS in general on clinicians’ knowledge, attitudes and clinical practice. Current teaching around FMS for both undergraduate and postgraduate learners also needs to be further developed.

## Conclusion

Undergraduate medical students have limited understanding of, and are sceptical over the existence of FMS. These attitudes are influenced by the 'hidden curriculum' and witnessing attitudes and actions of their clinical teachers. Students interpret a lack of formal curriculum teaching around FMS to mean that it is not serious and hence a low priority. Encountering a patinet, friend or family member with FMS can increase knowledge and lead to altered perception of the condition.

Teaching and learning about FMS needs to be consistent to improve knowledge and attitudes of clinicians. Undergraduate students should be exposed to patients with FMS so that they better understand patients with FMS.

## Abbreviations

CWP: Chronic Widespread Pain
FMS: Fibromyalgia syndrome
GPs: General Practitioners
IBS: Irritable Bowel Syndrome
MUS: Medically unexplained symptoms
PBL: Problem Based Learning
